# The metastasis-associated protein MTA3 promotes cardiac repair by inhibiting the fibroblast to myofibroblast transition during fibrosis

**DOI:** 10.1016/j.jbc.2025.110448

**Published:** 2025-07-02

**Authors:** Xu Wang, Yihui Liu, Heng Liu, Mengfan Zhang, Lida Yang, Khuzin Dinislam, Hongbo Hu, Dan Xiao, Huan Yang, Ying Zhang

**Affiliations:** 1 Department of Pharmacology, College of Pharmacy, Harbin Medical University, Harbin, China; 2 The State Key Laboratory of Frigid Zone Cardiovascular Diseases (SKLFZCD), and the Key Laboratory of Cardiovascular Medicine Research, Ministry of Education, Harbin, China; 3 Department of Microbiology, School of Basic Medical Sciences, WU Lien-Teh Institute, Harbin Medical University, Harbin, China; 4 The Second Affiliated Hospital of Harbin Medical University, Harbin, China; 5 Heilongjiang Nursing College, Harbin, Heilongjiang, China; 6 Department of General Chemistry, Bashkir State Medical University, Ufa, Republic of Bashkortostan, Russia; 7 Zhengzhou Research Institute, Harbin Institute of Technology, Zhengzhou, Henan, China

**Keywords:** cardiac fibrosis, fibroblast, myofibroblast, MTA3, transformation

## Abstract

Cardiac fibrosis is a pathological hallmark of various cardiovascular disorders. Accumulating evidence has demonstrated that fibroblasts transform into myofibroblasts during the occurrence of cardiac fibrosis, but the mechanism remains incompletely understood. This study aims to investigate the relevance of MTA3 as a potential therapeutic target for cardiac fibrosis. The myocardial infarction model was established by ligating the left coronary artery of C57BL6 mice, and myocardial fibrosis was measured by cardiac ultrasound and Sirius red staining of myocardium. MTA3 overexpression plasmid was constructed and transfected into primary fibroblasts, immunofluorescence, Western blot and qRT-PCR were used to detect the expression of MTA3, **α**-SMA, and Collagen I. RNAi was used to interfere with the downstream potential target gene E2F1. SB203580, a specific inhibitor of p38 MAPK, reduced the levels of phosphorylated p38 MAPK (p-p38) by inhibiting p38 MAPK activity, and allowed assessment of MTA3-induced fibroblast to myofibroblast transformation. The expression of MTA3 was reduced in fibrotic myocardium. Overexpression of MTA3 could restore cardiac function. During the transformation process of cardiac fibroblasts into myofibroblasts, the expression of MTA3 was downregulated. After overexpression of MTA3, the mRNA and protein levels of **α**-SMA and Collagen I were significantly reduced. When E2F1 was disrupted, the mRNA and protein levels of **α**-SMA and Collagen I were downregulated. Inhibition of p-p38 MAPK expression by SB203580 ameliorated myocardial fibrosis. MTA3 regulates the transformation of fibroblast into myofibroblast by p38 MAPK-E2F1 signaling pathway, and MTA3 may become a potential target for treating cardiac fibrosis.

Cardiac fibrosis refers to the excessive accumulation of collagen fibers in myocardial tissue and the pathological changes in collagen content and concentration (1, 2, 3). Previous studies have pointed out that the occurrence of many cardiovascular system diseases, such as hypertension, atherosclerosis, myocardial infarction, and heart failure, is closely related to the occurrence of myocardial fibrosis (4, 5, 6). In recent years, Numerous studies have demonstrated that cardiac fibroblasts (CFs) proliferate and differentiate in the early stages of cardiac injury (7, 8, 9). Myocardial remodeling predominantly results from the accumulation of extracellular collagen (10). At the same time, in the development process of cardiac fibrosis, the trans differentiation of cardiac fibroblasts into myofibroblasts (MFs) is a crucial reason (11).

Tumor metastasis-associated protein 3 (MTA3) is a member of the metastasis-associated protein (MTA) family and is the focus of current scientific research (12, 13). It is an important component of the nucleosome remodeling and histone deacetylase complex (14). The acetyl complex can control protein activity expression by regulating protein acetylation. At the same time, this regulation of acetylation and deacetylation can change the transmission process of various signaling pathways, thereby producing a variety of biological processes (15). MTA3 has been widely studied in tumors (16, 17), but its role in the cardiovascular system has been rarely reported. Studies have shown that the expression of MTA3 is significantly downregulated when fibrosis occurs (18). Therefore, in-depth research on the regulatory effect and mechanism of MTA3 on myocardial fibrosis will provide more effective and accurate approaches for related research on cardiovascular system diseases. MTA3 is expected to become a novel biological indicator and treatment target for clinical disease. E2F transcription factor 1 (E2F1) is a member of the cell cycle-related transcription factor family and can induce epithelial-mesenchymal transition in lung cancer and osteosarcoma cells (19, 20). In addition, knocking down the expression of E2F1 in rat cardiomyocytes can significantly increase the level of apoptosis (21). We speculate that E2F1 is involved in the process of myocardial fibrosis.

Our study used a well-established murine myocardial infarction model of myocardial infarction and primary cardiac fibroblasts from neonatal mice to explore how MTA3 induces the transformation of fibroblast-to-myofibroblast transition (FMT) by regulating downstream E2F1 during cardiac fibrosis. It provides an important theoretical basis for revealing the mechanism of myocardial fibrosis.

## Results

### MTA3 expression is significantly downregulated in fibrotic myocardial tissue

**Figure 1:**
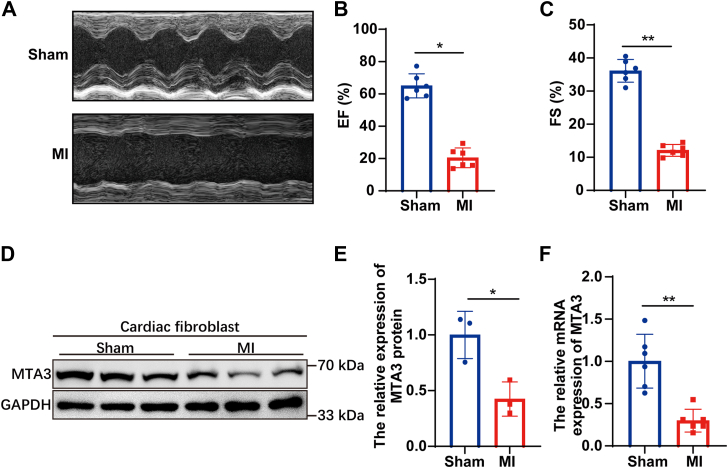
**MTA3 expression is significantly reduced in myocardial infarction mice.***A–C*, cardiac ultrasound showing that the cardiac function of the myocardial infarction (MI) model mice reduces significantly. *D* and *E*, Western blot and quantification of MTA3 protein level in cardiac fibroblasts from sham and myocardial infarction mice. *F*, qRT-PCR analyses showing MTA3 mRNA levels are significantly down-regulated in mice with myocardial infarction (∗*p* < 0.05, ∗∗*p* < 0.01).

To study the role of MTA3 in cardiac fibrosis, we first analyzed cardiac MTA3 expression in mice subjected to myocardial infarction (MI) through ligation of the left anterior descending coronary artery. 4 weeks after ligation, ejection fraction (EF) (%) and FS (%) values were significantly reduced compared with sham group (Fig. 1, *A*–*C*). To further investigate the specific mechanism by which MTA3 regulates myocardial fibrosis, we first compared the expression differences of MTA3 in primary cardiomyocytes and fibroblasts from myocardial infarction (MI) mice and sham controls. Our results demonstrated that the protein and mRNA levels of MTA3 were significantly reduced post-MI in cardiac fibroblasts (Fig. 1, *D*–*F*). In contrast, there was no significant change in the expression level of MTA3 protein in cardiomyocytes after myocardial infarction (Fig. S1, *A*and *B*). Our results suggested that MTA3 played an important role in cardiac fibrosis process.

### Overexpression of MTA3 reverses cardiac fibrosis in mice

**Figure 2:**
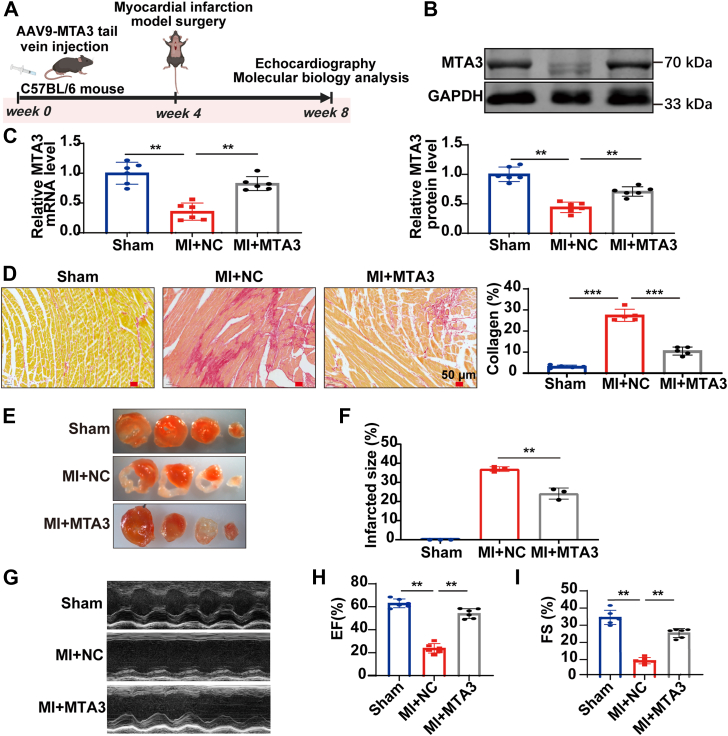
**Overexpression of MTA3 reverses cardiac fibrosis in mice with myocardial infarction**. *A*, schematic for AVV9-MTA3 administration and myocardial infarction model construction. *B* and *C*, MTA3 is successfully overexpressed at the protein and mRNA levels detected by Western blot and qRT-PCR. *D*, Sirius red staining showing that overexpression of MTA3 reverses collagen deposition in myocardial fibrosis tissue. *E* and *F*, Triphenyltetrazolium chloride staining of heart sections showing that overexpression of MTA3 reduces infarcted area compared to controls. *G–I*, echocardiography showing that overexpression of MTA3 significantly improves cardiac function in mice with myocardial infarction (∗∗*p* < 0.01, ∗∗∗*p* < 0.001).

To further clarify the correlation between MTA3 and cardiac fibrosis, we overexpressed MTA3 in cardiac fibroblasts specifically by adeno-associated virus in mice with myocardial infarction (Fig. 2, *A*–*C*). Sirius red staining revealed that mice in the myocardial infarction model group developed significant fibrosis, and overexpression of MTA3 significantly alleviated cardiac fibrosis (Fig. 2*D*). To quantitatively assess myocardial infarction size, we performed triphenyl tetrazolium chloride (TTC) staining on whole-heart sections. The results demonstrated that MTA3 overexpression significantly reduced infarct area compared to controls (Fig. 2, *E* and *F*). Furthermore, immunofluorescence analysis of cardiac tissues revealed that MTA3 overexpression markedly attenuated cardiac fibrosis (Fig. S2*A*).

The cardiac function of mice was evaluated by echocardiography, and it was found that the cardiac function of mice with a myocardial infarction model after overexpression of MTA3 was significantly restored, and the EF% and FS% values were significantly increased (Fig. 2, *G*-*I*). The above results indicate that MTA3 may become a novel target for treating cardiac fibrosis.

### MTA3 expression is downregulated during the transformation of cardiac fibroblasts into myofibroblasts

**Figure 3:**
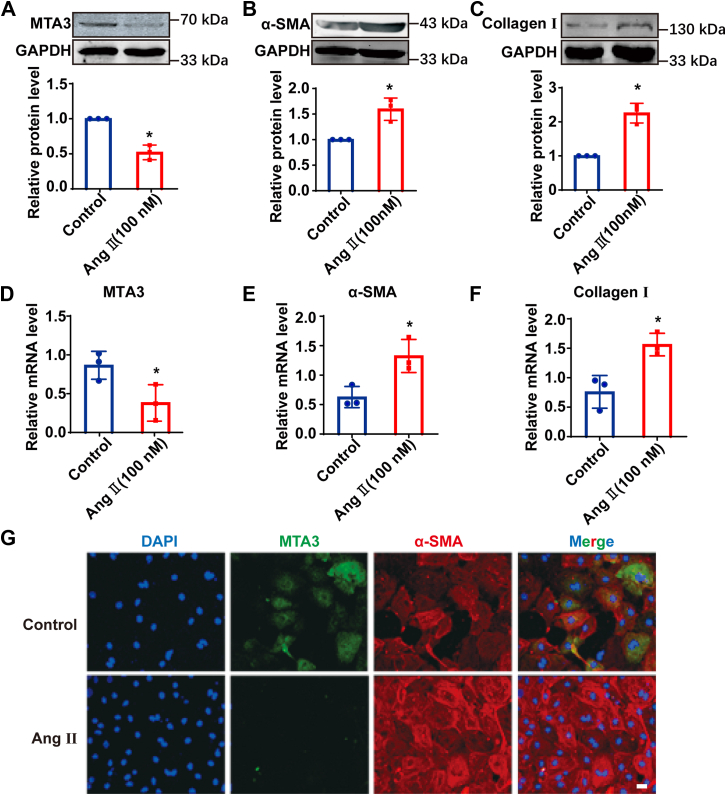
**The establishment of cell model of cardiac fibrosis, and the expression of MTA3 on AngⅡ induced fibroblast-to-myofibroblast transition.***A–C*, MTA3, α-SMA and Collagen Ⅰ protein expression are detected by Western blot in control and AngⅡ treated 24 h cardiac fibroblast. *D–F*, qRT-PCR analysis of mRNA expression level of MTA3, α-SMA, and Collagen Ⅰ in control and AngⅡ-treated 24 h cardiac fibroblast. *G*, protein expression of α-SMA (*red*) and MTA3 (*green*) was assessed by immunofluorescence staining. Nuclei were visualized by DAPI (*blue*). Scale bar = 20 μm. Each data point represents one cell well, and each well contains cells derived from multiple hearts (∗*p* < 0.05).

Studies have shown that during the process of TGFβ1-induced myocardial fibrosis, the expression of MTA3 protein is significantly down-regulated. We used AngII to treat primary cardiac fibroblasts for 24 h to induce a cell model of cardiac fibrosis. From Figure 3, *A* and *D*, the protein and mRNA expression levels of MTA3 decreased significantly compared with the control group after 24 h of AngII treatment. Correspondingly, the protein and mRNA expression levels of α-SMA and Collagen Ⅰ increased significantly compared with the control group (Fig. 3, *B*, *C*, *E,* and *F*). Fibroblasts were double-stained with MTA3 and α-SMA by immunofluorescence. Compared with the control group, the expression of MTA3 was significantly reduced after AngII treatment, while the expression of α-SMA was significantly increased compared with the control group, and the number of cells was increased (Fig. 3*G*).

The above results show that AngII can induce the transformation of cardiac fibroblasts into myofibroblasts, and the expression of MTA3 is significantly downregulated during this process.

### MTA3 overexpression can effectively inhibit the transformation of cardiac fibroblasts into myofibroblasts

**Figure 4:**
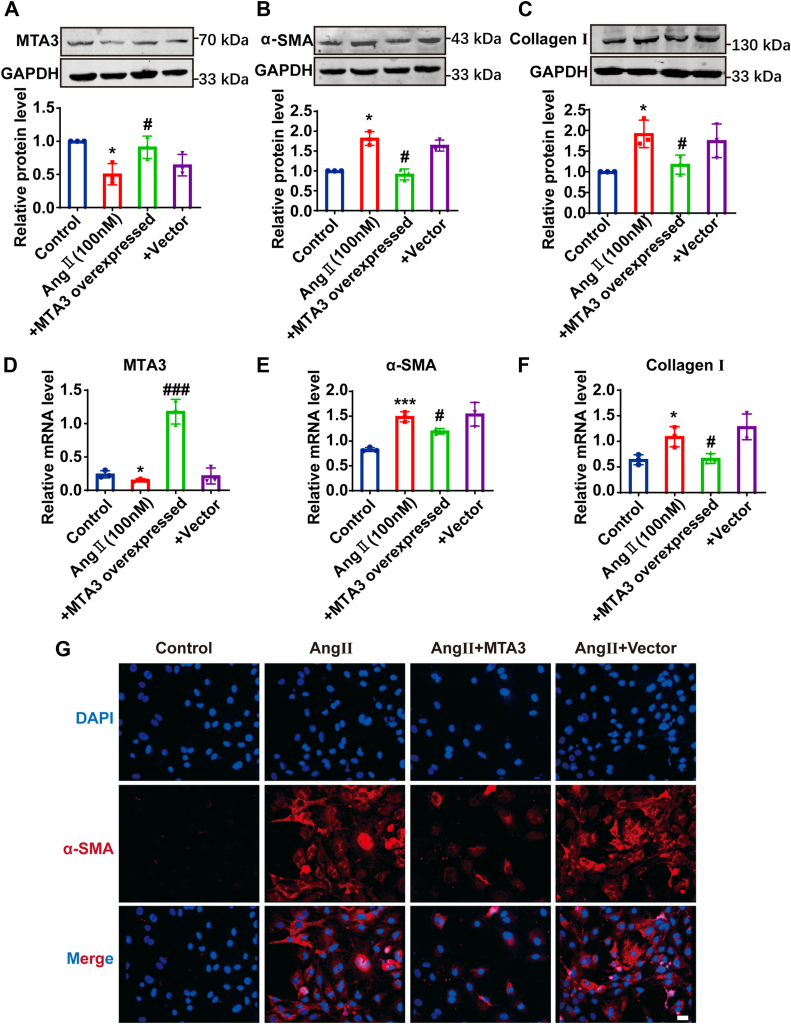
**Overexpression of MTA3 can effectively inhibit fibroblast-to-myofibroblast transition.***A–C*, MTA3, α-SMA, and Collagen Ⅰ protein expression was detected by Western blot in cardiac fibroblasts. Grouped into control, AngⅡ treated 24 h, AngⅡ treated 24h and transfection with plasmid vector of MTA3 overexpression, AngⅡ treated 24 h and transfection with empty plasmid (*D–F*), qRT-PCR analysis of mRNA expression level of MTA3, α-SMA, and Collagen Ⅰ in each cell group. *G*, protein expression of α-SMA (*red*) was assessed by immunofluorescence staining. nuclei were visualized by DAPI (*blue*). Scale bar = 20 μm. Each data point represents one cell well, and each well contains cells derived from multiple hearts (∗*p* < 0.05, ∗∗∗*p* < 0.001 *versus* Control, n = 3, ^#^*p* < 0.05, ^###^*p* < 0.001 *versus* AngⅡ treated 24 h and transfection with empty plasmid).

To explore whether MTA3 is involved in the process of converting CFs into MFs, primary cardiac fibroblasts were transfected with a plasmid vector to overexpress MTA3. The results in Figure 4, *A* and *D* show that overexpression of MTA3 can significantly increase the mRNA and protein levels of MTA3, and the increase in the mRNA level is significantly higher than the protein level. Correspondingly, the protein and mRNA expression levels of α-SMA and Collagen Ⅰ, which were the markers for the cardiac fibroblasts’ transformation, were significantly downregulated after MTA3 overexpression (Fig. 4, *B*, *C*, *E,* and *F*). To further prove this regulatory role of MTA3, immunofluorescence staining of α-SMA was performed. The result showed that MTA3 overexpression could effectively inhibit the increase in the fluorescence intensity of α-SMA induced by AngII (Fig. 4*G*). Collectively, these results indicated that upregulation of MTA3 expression can effectively inhibit the transformation of CFs into MFs.

### Knocking down MTA3 in cardiac fibroblasts promotes its transformation into myofibroblasts

**Figure 5:**
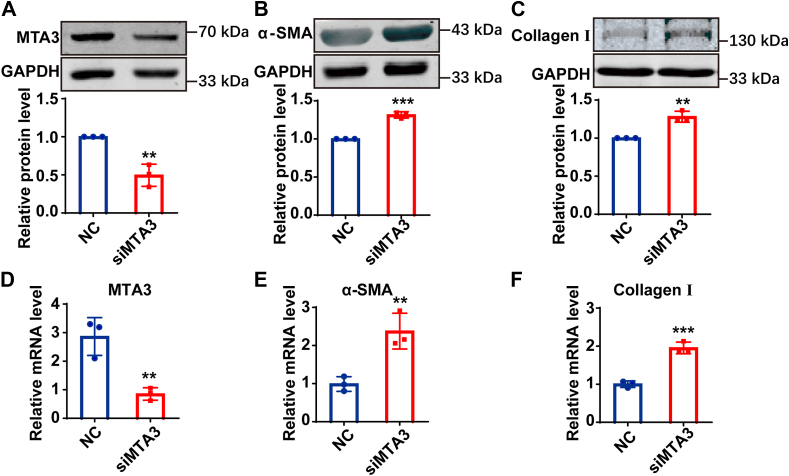
**The knockdown of MTA3 in cardiac fibroblast can promote fibroblast-to- myofibroblast transition.***A–C*, MTA3, α-SMA and Collagen Ⅰ protein expression in normal cardiac fibroblast and which were transfected by siRNA. *D–F*, qRT-PCR analysis of mRNA expression level of MTA3, α-SMA and Collagen Ⅰ in normal cardiac fibroblast and which were transfected by siRNA. Each data point represents one cell well, and each well contains cells derived from multiple hearts (∗∗*p* < 0.01, ∗∗∗*p* < 0.001).

To further determine the role of MTA3 in the transformation of CFs into MFs, RNA interference technology was used to knock down MTA3 in cardiac fibroblasts. The protein level of MTA3 decreased by approximately 50%, while the mRNA level decreased by approximately 70% (Fig. 5, *A* and *D*). The expression of α-SMA and Collagen Ⅰ, classical markers for the transformation of cardiac fibroblasts into myofibroblasts was also detected. As expected, the protein and mRNA expression levels of α-SMA and Collagen Ⅰ were significantly up-regulated after MTA3 was knocked down (Fig. 5, *B*, *C*, *E,* and *F*). Altogether, the above results show that knocking down MTA3 in cardiac fibroblasts promotes its transformation into myofibroblasts, and MTA3 indeed plays a crucial role in the process of cardiac fibrosis.

### MTA3 inhibits the expression of E2F1, and knocking down E2F1 reverses the inhibitory effect of MTA3 on cardiac fibroblasts transformation

**Figure 6:**
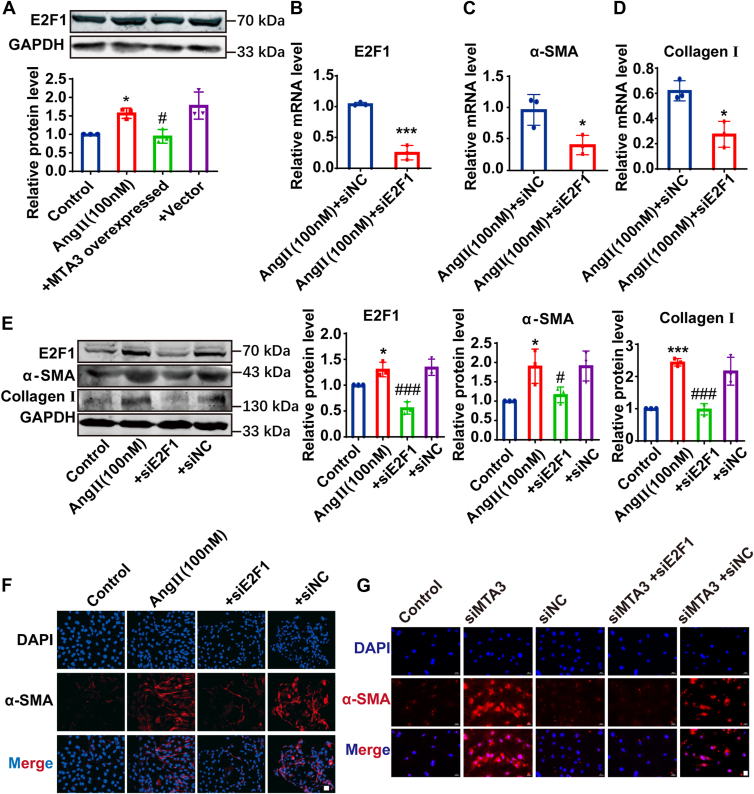
**MTA3 can inhibit the expression of transcription factor E2F1, while the knockdown of E2F1 in cardiac fibroblasts can inhibit fibroblast-to-myofibroblast transition in the cellular level**. *A*, E2F1 protein expression was detected by Western blot in cardiac fibroblasts. Grouped into control, AngⅡ treated 24 h, AngⅡ treated 24 h and transfection with plasmid vector of MTA3 overexpression, AngⅡ treated 24 h, and transfection with empty plasmid. *B–D*, E2F1, α-SMA, and CollagenⅠ mRNA expression levels detected by qRT-PCR in cardiac fibroblasts. *E*, E2F1, α-SMA, and Collagen Ⅰ protein expression levels detected by Western blot in cardiac fibroblasts. Grouped into control, AngⅡ treated 24 h, AngⅡ treated 24 h and transfection with siE2F1, AngⅡ treated 24 h and transfection with negative control. *F*, immunofluorescence staining of α-SMA (*red*) and DAPI (*blue*) in cardiac fibroblast interfered with MTA3 or MTA3 and E2F1, scale bar = 20 μm. G. Immunofluorescence staining of α-SMA (*red*) and DAPI (*blue*) in cardiac fibroblast interfered with siMTA3 , siNC, siMTA3+siE2F1 and siMTA3+siNC, scale bar = 20 μm. Each data point represents one cell well, and each well contains cells derived from multiple hearts (∗*p* < 0.05, ∗∗∗*p* < 0.001 *versus* Control, n = 3, ^#^*p* < 0.05, ^###^*p* < 0.001 *versus* AngⅡ treated 24 h and transfection with empty plasmid).

The transcription factor E2F1 affects the progression of the cell cycle (22), plays an important role in the excessive increase of lung fibroblasts in pulmonary fibrosis (23, 24). We speculate that E2F1 may regulate the transformation of cardiac fibroblasts into myofibroblasts during cardiac fibrosis. The downregulation of MTA3 may promote the expression of some transcription factors. In this study, the expression of E2F1 protein was significantly increased after 24h of AngII treatment compared with the control group, and overexpression of MTA3 could significantly inhibit the expression of E2F1 protein (Fig. 6*A*). The above results indicate that E2F1 may be a key downstream transcription factor for MTA3 in regulating the transformation of fibroblasts into myofibroblasts. Figure 6*B* shows that based on 24 h of AngII induction, transfection of E2F1 interfering RNA into fibroblasts can effectively inhibit the expression of E2F1 protein. The protein and mRNA expression levels of α-SMA and Collagen Ⅰ were significantly down-regulated compared with the control group after knocking down E2F1 in AngII-treated cardiac fibroblasts (Fig. 6, *C*–*E*). The immunofluorescence results also indicated that interfering with E2F1 could reverse AngII-induced cardiac fibroblast transformation (Fig. 6*F*). This confirms that knocking down E2F1 at the cellular level can inhibit the conversion of CFs into MFs.

Consistently, overexpression of MTA3 in fibroblasts of cardiac tissue significantly attenuated MI-induced upregulation of E2F1 (Fig. S3, *A*–*C*). Furthermore, in cardiac fibroblasts, MTA3 knockdown (siMTA3) markedly enhanced fibrotic responses as evidenced by increased α-SMA expression, whereas concomitant E2F1 knockdown (siE2F1) effectively reversed these pro-fibrotic effects (Fig. 6*G*). The above results indicate that MTA3 suppresses fibroblast activation through E2F1-dependent mechanisms.

### MTA3 regulates the expression of E2F1 through the p38 MAPK signaling pathway

**Figure 7:**
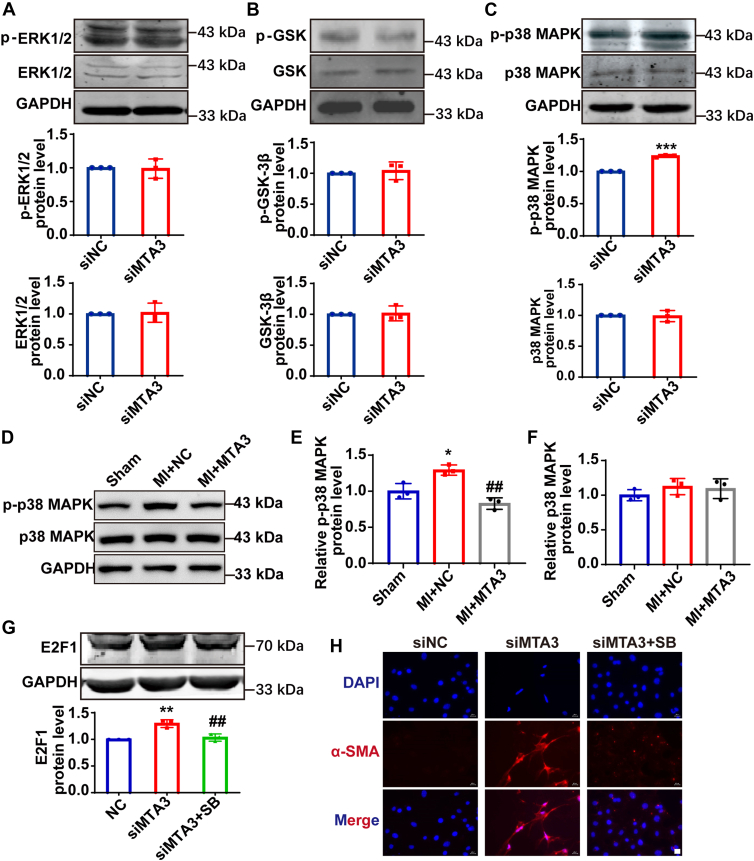
**MTA3 regulates E2F1 expression through the p38 MAPK signaling pathway.***A–C*, ERK1/2, GSK-3β and p38 MAPK protein and their phosphorylated form expression levels in normal cardiac fibroblast and which transfection with siRNA detected by Western blot. *D–F*, Western blot and quantification of p-p38 MAPK and p38 MAPK protein levels in MTA3 overexpression mice compared with the control group after myocardial infarction. *G*, Western blot analysis of E2F1 expression in each group. Grouped into negative control, siMTA3, siMTA3 and p38 MAPK inhibitor (SB203580) treatment group. n = 3. *H*, immunofluorescence staining of α-SMA (*red*) and DAPI (*blue*) in cardiac fibroblast interfered with MTA3 and then treated with saline or SB203580 (a specific inhibitor of p38 MAPK), scale bar = 20 μm. Each data point represents one cell well, and each well contains cells derived from multiple hearts (∗*p* < 0.05, ∗∗*p* < 0.01 *versus* negative control, n = 3, #*p* < 0.05 *versus* transfection with siMTA3).

To explore the signaling pathways through which MTA3 regulates E2F1 and thereby affects the transformation process from fibroblasts into myofibroblasts, the expression of phosphorylated proteins of signaling pathway molecules that are highly related to myocardial fibrosis was detected. After transfecting MTA3 interfering RNA into cardiac fibroblasts, the expression levels of ERK1/2 and GSK3β and their phosphorylated form had no significant difference (Fig. 7, *A* and *B*). Though the p38 MAPK protein level remained unchanged, the expression of phosphorylated p38 MAPK protein was significantly increased after MTA3 was knocked down under normal and myocardial infarction conditions (Fig. 7, *C*–*F*). Subsequently, we inhibited the p38 signaling pathway using SB203580, a specific inhibitor of p38 MAPK. This treatment significantly reduced the catalytic activity of p38 MAPK, as evidenced by the decreased levels of its downstream targets, while the expression of total p38 MAPK protein remained unchanged in MTA3-knockdown cells. Next, we detected the expression of E2F1 protein after applying SB203580. The results showed that the expression of E2F1 protein was significantly increased after MTA3 was knocked down, while the situation could be effectively alleviated after SB203580 treatment (Fig. 7*G*).

Immunofluorescence staining demonstrated that MTA3 knockdown (siMTA3) markedly increased α-smooth muscle actin (α-SMA) expression, an effect that was significantly reversed by treatment with a specific MAPK phosphorylation inhibitor (Fig. 7*H*). The above results indicate that MTA3 regulates E2F1 mainly through the p38 MAPK signaling pathway. In conclusion, MTA3 regulates cardiac fibrosis through the p38 MAPK-E2F1 axis.

## Discussion

Myocardial tissue injury initiates a cascade of inflammatory signaling that activates resident cardiac fibroblasts, triggering their phenotypic transformation into myofibroblasts (25). These activated cells exhibit enhanced migratory capacity, facilitating their recruitment to sites of injury (26). While the molecular mechanisms governing cardiac fibrosis remain incompletely understood and certain aspects continue to be debated, accumulating evidence indicates that fibroblast-to-myofibroblast transition (FMT) constitutes a key pathological event driving excessive extracellular matrix deposition during fibrotic remodeling (27, 28).

Extensive investigations have characterized the oncogenic roles of MTA3 in various malignancies. Notably, Wu *et al.* demonstrated that MTA3 mediates chemoresistance in pancreatic ductal adenocarcinoma by promoting gemcitabine resistance through epigenetic regulation of drug metabolism pathways (29). Wang's research also showed that MTA3 can interact with HDAC to induce non-small cell lung cancer cell migration and invasion by targeting c-Myc and cyclin D1 (30). However, there are few reports on its role in the cardiovascular system. The results of this study showed that treatment with 100 nM of AngII for 24 h could successfully induce the transformation of cardiac fibroblasts into myofibroblasts. Using both *in vivo* animal models and *in vitro* cell culture systems, mRNA and protein expression levels of MTA3 were significantly downregulated compared with the control group. Overexpression of MTA3 could significantly reverse the cardiac function caused by myocardial infarction and inhibit the expression of myofibroblasts markers. The method of siRNA was also used to knock down MTA3 in CFs, which showed that MTA3 is indeed a key regulatory factor in the occurrence of FMT. These results are consistent with those of Xiao’s study (18) and Qin’s study (31). Also, we have conducted in-depth research on the specific mechanism of MTA3 regulating cell transformation.

Studies have confirmed that the E2F1 gene was involved in the proliferation process of renal interstitial cells and is especially closely related to the process of neurological fibrosis (32). P38 MAPK is an important member of the MAPK family, especially in cellular transcriptional and post-transcriptional regulatory processes (33). In this study, we found that overexpression of MTA3 in cardiac fibroblasts inhibited the expression of E2F1 protein. After inhibiting phosphorylated p38 MAPK with SB203580, a specific inhibitor of phosphorylated p38 MAPK, it was found that the expression of E2F1 was significantly down-regulated. These results indicate that MTA3 regulates E2F1 protein expression *via* the p38 MAPK signaling pathway. This study provides a preliminary characterization of p38 MAPK involvement, while the underlying mechanisms require further systematic investigation. We inhibited or overexpressed MTA3 and measured various corresponding indicators; the results suggested that MTA3 could inhibit the process of phenotypic conversion from CFs to MFs. However, the MAPK cascade reaction is an extremely complex, intertwined connection network. Therefore, to fully explain the impact of MTA3 on cardiac fibrosis, animal experiments need to be improved, and its specific molecular mechanism needs to be further explored.

**Figure 8:**
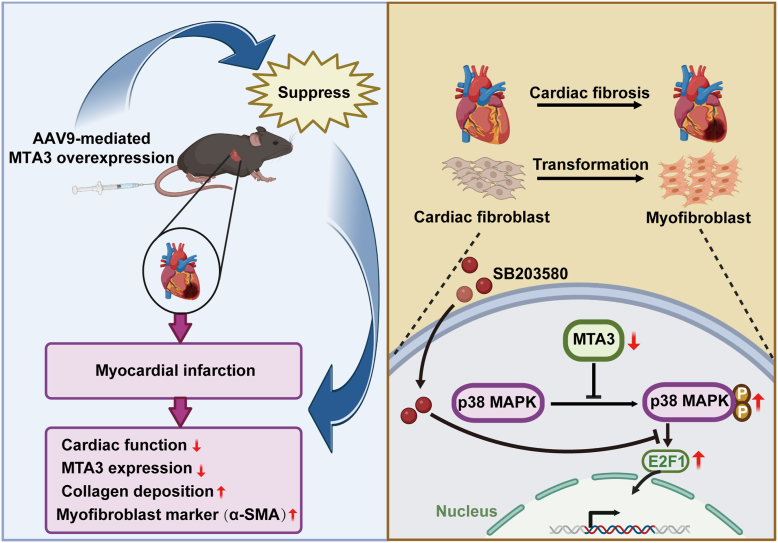
**Schematic diagram summarizing the mechanism.** AAV9-mediated MTA3 overexpression improves cardiac function after myocardial infarction. MTA3 supresses the fibroblast-to-myofibroblast transition during cardiac fibrosis through inhibiting p38 MAPK-E2F1 signaling pathway.

To sum up, we used MTA3 as the research object for the first time on CFs at the *in vivo* and *in vitro* levels. We found that AAV9-mediated MTA3 overexpression could inhibit cardiac fibrosis and improve cardiac function after myocardial infarction. We knocked down or overexpressed MTA3 in CFs, detected the changes in its phenotypic transformation, and concluded that MTA3 plays a role in cardiac fibrosis. Subsequently, we found the specific molecular mechanism of MTA3 in cardiac fibrosis by regulating the p38 MAPK-E2F1 signaling pathway (Fig. 8).

In our study, we observed that the lack of change in protein content in cardiomyocytes suggests that MTA3 may not have a direct effect on these cells. However, the significant impact on infarct size indicates that MTA3 likely exerts its influence upstream of fibrosis. This suggests that MTA3 may modulate signaling pathways or cellular processes that precede fibroblast activation and extracellular matrix deposition. Additionally, we have explored the role of MTA3 in cardiac fibrosis and its potential to modulate p38 MAPK activity. We propose several mechanisms through which MTA3 might influence p38 MAPK, including inhibiting upstream kinases like MKK3, MKK6, or MKK4, interacting with TAB1 to prevent p38 autophosphorylation, and modulating ZAP70 activity to affect p38α phosphorylation (34, 35). These hypotheses are based on current knowledge of p38 MAPK regulation and require further investigation. Future research will focus on elucidating these upstream mechanisms and verifying the proposed pathways to provide a more comprehensive understanding of MTA3's role in cardiac fibrosis. In conclusion, we proposed that MTA3-p38 MAPK-E2F1 axis may be novel targets for the prevention and treatment of cardiac fibrosis.

In this study, we found for the first time that MTA3 inhibits myocardial fibrosis by regulating the transformation of myocardial fibroblasts into myofibroblasts at the animal and cellular levels and revealed the detailed mechanism of the p38 MAPK-E2F1 signaling pathway. This study provides an important theoretical basis for understanding the causes of myocardial fibrosis during myocardial infarction and for developing therapeutic targets for myocardial fibrosis in the future.

## Experimental procedures

### Construction of a mouse myocardial infarction model

C57BL6 male mice were used to establish a myocardial infarction model by ligating the left anterior descending branch of their coronary artery. The mice were anesthetized by intraperitoneal injection of the prepared 2,22-tribromoethanol solution. Blunt-tipped curved forceps were used to open the mouse chest cavity and expose the heart. Subsequently, the left anterior descending branch of the coronary artery was quickly ligated with a 7-0 surgical suture. All animal experiments were approved by the Research Council of Harbin Medical University (41008211).

### Construction of MTA3 overexpression vector

MTA3 overexpression adeno-associated virus, and overexpression negative control adeno-associated virus were injected into the tail vein of mice. The virus used adeno-associated virus 9 (AAV9) as a vector and normal saline as a solvent. AAV9 serotype with a periostin (POSTN) promoter was employed to drive MTA3 overexpression, as POSTN is selectively activated in fibroblasts during cardiac fibrosis. We administered AAV9-POSTN-MTA3 *via* tail vein injection, with a dosage of 2 × 10^11^ GC (Genome containing particles) at the following time points (1): Initial injection at week 0 (2); Second injection at week 4, post-MI surgery. This protocol ensured sustained MTA3 expression throughout both acute and chronic remodeling phases. The myocardial infarction model was established 4 weeks after the first injection of the virus.

### Cardiac ultrasound

The cardiac function of mice was detected 4 weeks after myocardial infarction. The small animal Doppler ultrasound Vevo1100 imaging system (FUJIFILM Visual Sonics) was used to calculate the EF and left ventricular short-axis shortening (Fraction shortening, FS) to evaluate the cardiac function of mice.

### Sirius red staining

Soak the dried tissue slide in xylene for 10 min, add the prepared iron hematoxylin stain solution to the tissue, completely cover the tissue, and stain for 6 min. Rinse the slide with running water for 5 min. Add Sirius red stain solution to the surface of the tissue, stain for 20 min, and then dehydrate. Use a microscope to observe the tissue staining.

### Isolation and culture of neonatal mouse cardiac fibroblasts

Place the whole body of the suckling rat in a container outside the ultra-clean table for about 2 min, and then place it in an internal container for sterilization for 2 min. Then pinch the skin on the back of the suckling mouse and cut the skin on the chest to make the heart bounce out. Place in a centrifuge tube and add trypsin for digestion. Filtration and centrifugation are used to redisperse the precipitate with high-glucose Dulbecco's modified Eagle's medium (DMEM; Gibco; Thermo Fisher Scientific, Inc.) containing 10% fetal bovine serum and 1% penicillin/streptomycin to obtain fibroblasts and cardiomyocytes. Cardiac fibroblasts were isolated from neonatal rat hearts using differential adhesion: following enzymatic digestion, cells were plated for 1.5 h, after which non-adherent cardiomyocytes were removed. The adherent fibroblasts were cultured and used within 3 passages. We euthanized the suckling mice by cervical dislocation. To reduce the stress and pain of the mice, the experimental personnel must be trained and pass the assessment.

### Isolation and culture of adult mouse cardiac fibroblasts

Adult mouse cardiomyocytes (CMs) and CFs were isolated from the ventricles of wild-type mice. Briefly, after anesthesia, hearts were excised and perfused with EDTA buffer (130 mM NaCl, 5 mM KCl, 0.5 mM Na2HPO4, 10 mM HEPES, 10 mM Taurine, 10 mM D-glucose, 10 mM BDM, 5 mM EDTA, pH 7.8), followed by collagenase buffer (130 mM NaCl, 5 mM KCl, 0.5 mM Na2HPO4, 10 mM HEPES, 10 mM Taurine, 10 mM D-glucose, 1 mM MgCl2, 0.5 mg/ml collagenase II, 0.5 mg/ml collagenase IV, 0.05 mg/ml protease XIV) for digestion. The digested ventricles were dissociated into small pieces and transferred to a solution containing collagenase buffer. The cell suspension was filtered through a 100-μm filter to remove large tissue debris. The filtered cell suspension was centrifuged at 3000*g* for 10 min to pellet the cardiomyocytes (CMs). The supernatant containing non-cardiomyocyte cells was collected and centrifuged at 8000*g* for 10 min to pellet the CFs. The cardiomyocyte pellet was resuspended in culture medium and plated on laminin-coated dishes. The fibroblast pellet was further purified using CD45 MicroBeads (Miltenyi Biotec) according to the manufacturer's instructions to deplete immune cells (36).

### Cell transfection

When the density of fibroblasts reaches 50%-80%, the cells are starved for 6 h in serum-free culture medium and transfected with plasmid, negative control, Lipofectamine 2000 (Invitrogen; ThermoFisher Scientific, Inc) and other reagents respectively, using DMEM medium (containing 10% FBS), incubate for 24 h for the next step. siMTA3 sense CCG CAG AAG CCG AGA GCA A, antisense UUG CUC UCG GCU UCU GCG G. siE2F1 sense CCA AGA AGU CCA AGA AUC A, antisense UGA UUC UUG GAC UUC UUG G.

### Western blot

Cells were lysed at a ratio of Radio Immunoprecipitation Assay (RIPA) to protease inhibitors (Beyotime Biotechnology) of 100:1, and total cellular protein was extracted. Determine total cellular protein by BCA method according to the instructions of the Beyotime kit (Beyotime Biotechnology). SDS-polyacrylic acid amine gel (Epizyme Biotechnology, China) electrophoresis, add the processed protein sample and Marker, and conduct constant voltage electrophoresis. Connect the transfer electrode in order and set the constant current to 300 mA, and transfer the protein to nitrocellulose (NC) membrane (Millipore, MA). Add 1.5 ml of antibodies prepared in PBS respectively, and immunoblots were visualized using Odessey Infrared Imaging system (LI-COR Inc) (37, 38). Immunoblots were quantified using NIH Image J software. GAPDH was used as a protein-loading control. All antibodies used in this study were commercially available, and their details are as follows:Anti-MTA3 (Rabbit monoclonal Cat# ab176346; Abcam); Anti-α-SMA (alpha smooth muscle actin) (Rabbit polyclonal, Cat# ab5694; Abcam); Anti-Collagen I (Rabbit monoclonal, Cat# ab138492; Abcam); Anti-E2F1 (rabbit monoclonal, Cat# ab314311; Abcam); Anti-ERK1/2 (Rabbit polyclonal, Cat# ab17942; Abcam); Anti-Phospho-ERK1/2 (Thr202/Tyr204) (Rabbit monoclonal, Cat# ab278538; Abcam);Anti-GSK3β (Rabbit monoclonal, Cat# ab32391; Abcam);Anti-Phospho-GSK3β (Ser9) (Rabbit polyclonal, Cat# ab107166; Abcam);Anti-MAPK (Rabbit polyclonal, Cat# 308333; Abcam);Anti-Phospho-MAPK (Thr180/Tyr182) (Rabbit polyclonal Cat# 9211; Cell Signaling Technology).

### Immunofluorescence

Digest the cells in the culture flask, place them in a 24-well plate for culture, and process the cells until the growth density reaches about 60% to 75%. Next, fix the cells with 4% paraformaldehyde for 15 min. Use freshly prepared. The cell surface was washed with PBS, penetrated with 0.1% Triton for 30 min, washed with PBS, and blocked with goat serum for 1 h. Incubate with α-smooth muscle actin (α-SMA) primary antibody (1:100) overnight, treat with fluorescently labeled secondary antibody for 1 hour, and stain with DAPI (1:100) for half an hour at 20 °C. Each step in the middle needs to be washed three times with PBS. The time interval between two cleanings is 5 minutes, and the confocal microscope collects images.

### qRT-PCR

After cell treatment, RNA was extracted, and total RNA was extracted from cells using Trizol (Invitrogen), chloroform, isopropyl alcohol, 75% ethanol, and other reagents. The RNA concentration was determined, the loading volume of RNA was calculated, and reverse transcription was performed. The reverse transcription of RNA was performed using ReverTra Ace qPCR RT Kit (Toyobo) with a system of 10 μl. RT-PCR was quantified using the SYBR GREEN fluorescent dye method by SYBR Green Master Mix (Toyobo), and GAPDH was used as an internal parameter to calculate Ct and RQ values.

### Triphenyl tetrazolium chloride staining

The infarct size was evaluated using a TTC staining kit (Solarbio, G3005). The cardiac tissue was sliced continuously along the longitudinal axis. Subsequently, the samples were immersed in 2% TTC solution at 37 °C for 15 min. Images were then captured by stereomicroscopy (Zeiss Stemi 508, Germany), and quantitative analysis was conducted by Image J software.

### Statistical analysis

The data obtained from the experiment were expressed as mean ± Standard Deviation (mean ± SD). An unpaired *t* test was used to compare the difference between the two groups. One-way ANOVA was used to compare the differences between multiple groups. GraphPad Prism 8.0 software was used for statistical analysis. *p* < 0.05 was used as the significant difference standard for determining statistical significance.

## Data availability

All data used during the current study are available from the corresponding author on reasonable request.

## Supporting information

This article contains supporting information.

## Conflict of interest

The authors declare that they have no conflicts of interest with the contents of this article.
