# Supplementary material for: The metastasis-associated protein MTA3 promotes cardiac repair by inhibiting the fibroblast to myofibroblast transition during fibrosis
Source: J Biol Chem. 2025 Jul 2;301(8):110448. doi: 10.1016/j.jbc.2025.110448 (PMC12319557; doi:10.1016/j.jbc.2025.110448)
Supplement: Supplementary data [file mmc1.docx]

**Supplementary Materials for**

**The metastasis-associated protein MTA3 promotes cardiac repair by inhibiting the fibroblast to myofibroblast transition during fibrosis**

Xu Wang^1,2#^, Yihui Liu^3#^, Heng Liu ^1^, Mengfan Zhang^1^, Lida Yang^4^, Khuzin Dinislam^1,5^, Hongbo Hu ^3^, Dan Xiao^6^, Huan Yang^1^, Ying Zhang^7^*

1. Department of Pharmacology, College of Pharmacy, Harbin Medical University, Harbin 150081, China. The State Key Laboratory of Frigid Zone Cardiovascular Diseases (SKLFZCD), and the Key Laboratory of Cardiovascular Medicine Research, Ministry of Education, Harbin 150081, China.

2. Department of Microbiology, School of Basic Medical Sciences, WU Lien-Teh Institute, Harbin Medical University, Harbin 150081, China.

3. The Second Affiliated Hospital of Harbin Medical University, Harbin 150086, China.

4. Heilongjiang Nursing Collage, Harbin 150086, Heilongjiang, China.

5. Bashkir State Medical University, Department of General Chemistry, Ulitsa Lenina, 3, Ufa, Republic of Bashkortostan, Russia.

6. Zhengzhou Research Institute, Harbin Institute of Technology, Zhengzhou, Henan 450007, China.

7. The First Affiliated Hospital of Jinan University, Guangzhou 510632, Guangdong, China.

Xu Wang^#^and Yihui Liu^#^contributed equally to this work.

*Corresponding author: [zhangying@hrbmu.edu.cn](mailto:zhangying@hrbmu.edu.cn)

Corresponding address：Department of Pharmacology, College of Pharmacy, Harbin

Medical University, Harbin baojian road No.157, 150081, China.


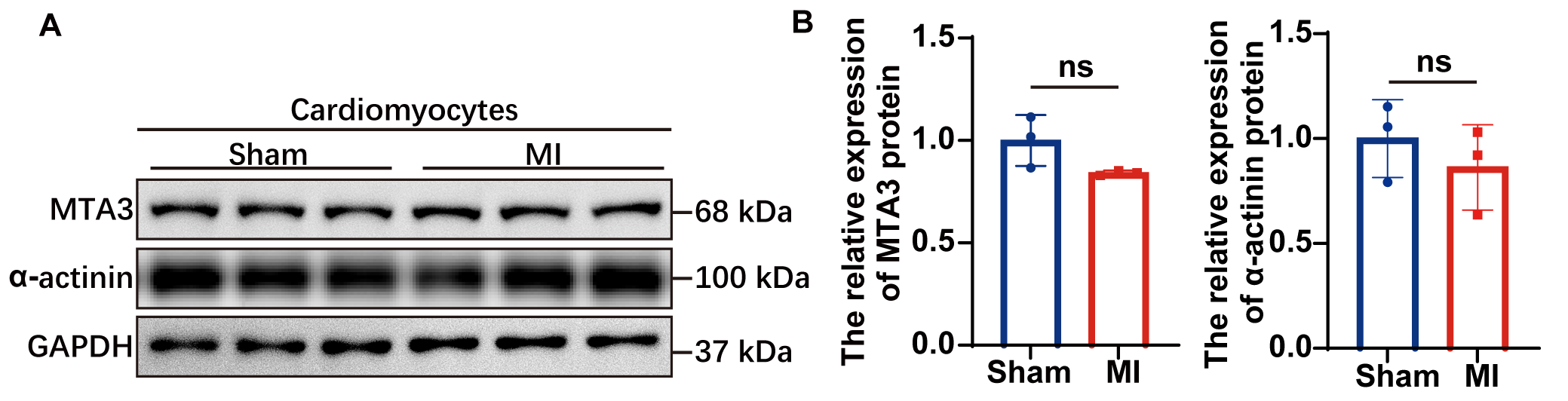


**Figure S1. MTA3 protein level has no significant change in cardiomyocytes after myocardial infarction**

(A-B) Western blot and quantification of MTA3 protein level in isolated cardiomyocytes at 4 weeks after myocardial infarction surgery or Sham. ns, no significance.


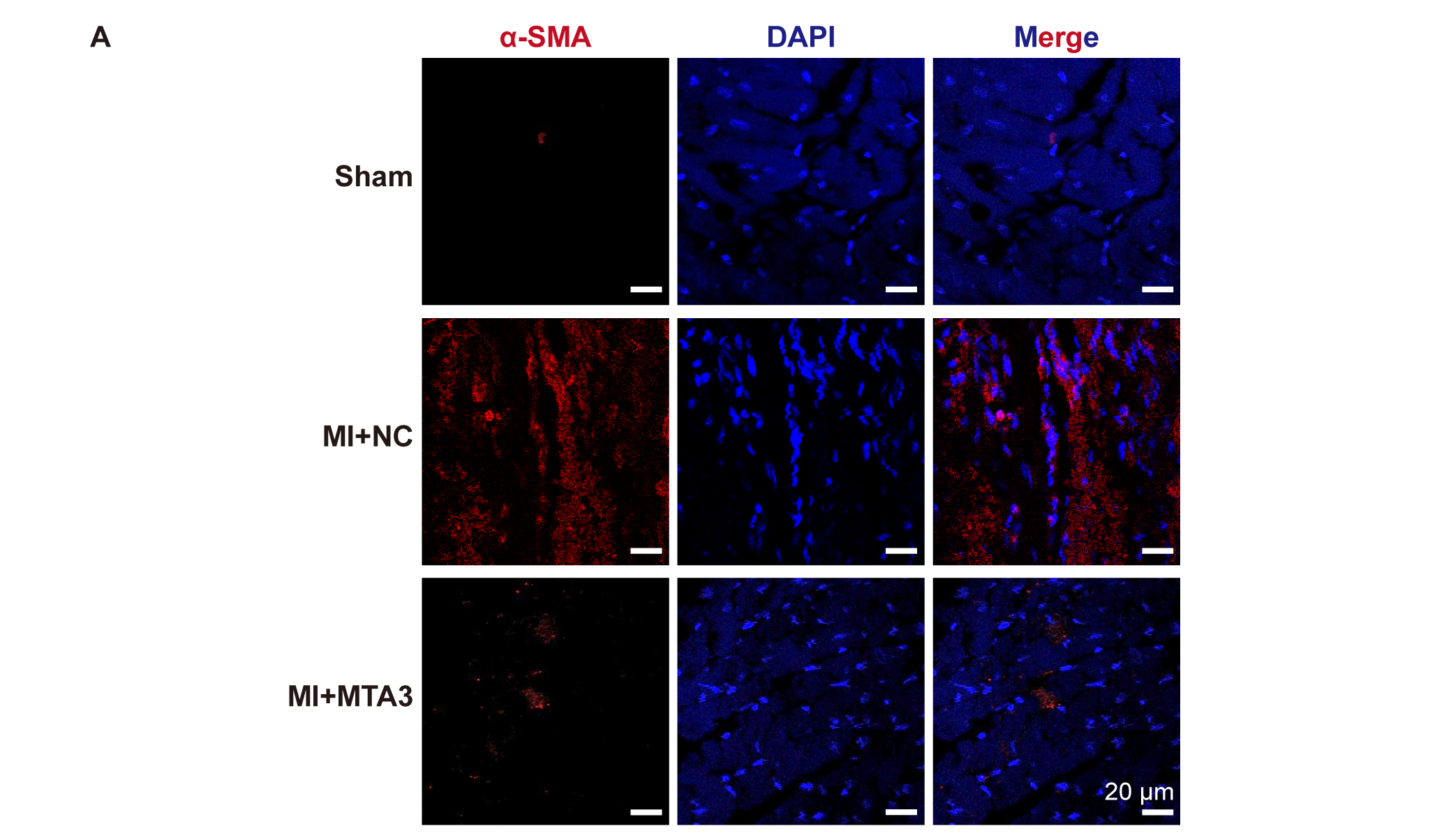


**Figure S2. Overexpression of MTA3 reverses cardiac fibrosis in mice with myocardial infarction**

(A) Immunofluorescence staining of α-SMA (red) and DAPI (blue) in the infarcted areas of mice at 4 weeks after myocardial infarction, scale bar = 20 μm.


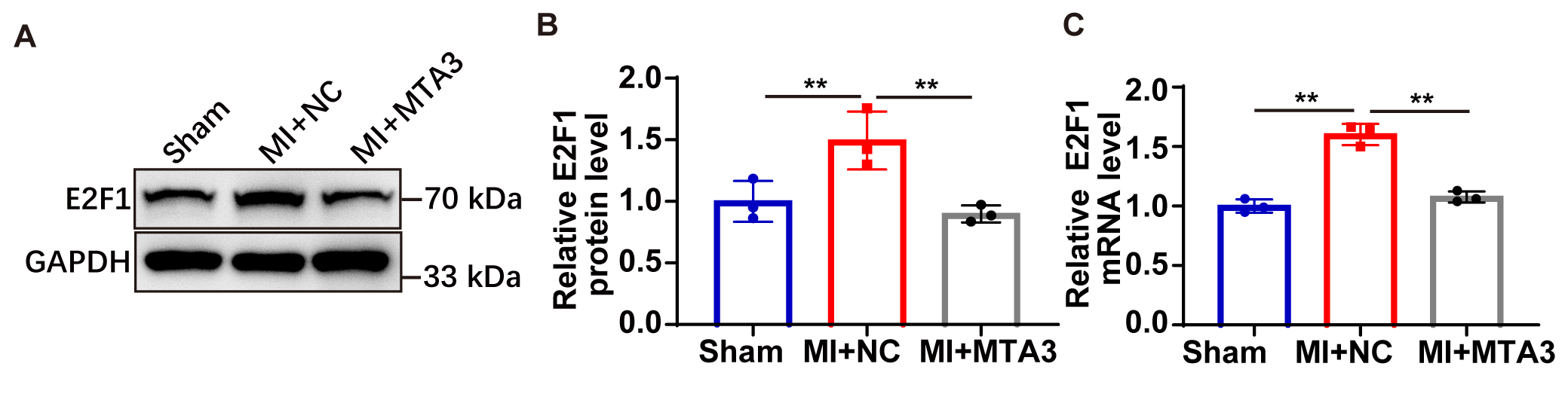


**Figure S3. E2F1 is a key downstream transcription factor of MTA3 in regulating the transformation of fibroblasts into myofibroblasts.**

(A-B) Western blot and quantification of E2F1 protein level in MTA3 overexpression mice compared with the control group after myocardial infarction. (C) E2F1 mRNA level in MTA3 overexpression mice compared with the control group after myocardial infarction detected by qRT-PCR (***P*<0.01).
